# Synergistic enhancement of production of proinflammatory cytokines of human peripheral blood monocytes by anti-Sm and anti-RNP antibodies

**DOI:** 10.1371/journal.pone.0209282

**Published:** 2018-12-20

**Authors:** Yu Matsueda, Yoshiyuki Arinuma, Tatsuo Nagai, Shunsei Hirohata

**Affiliations:** Department of Rheumatology and Infectious Diseases, Kitasato University School of Medicine, Kanagawa, Japan; Instituto Nacional de Ciencias Medicas y Nutricion Salvador Zubiran, MEXICO

## Abstract

The present study was performed to elucidate the roles of serum anti-Sm antibodies in the pathogenesis of systemic lupus erythematosus (SLE). Highly purified peripheral blood monocytes obtained from healthy donors were cultured in the presence of monoclonal anti-Sm antibody (anti-Sm mAb), monoclonal anti-U1-RNP antibody (anti-RNP mAb) or control murine IgG1 or IgG3. After various periods of incubation, levels of IL-6 and TNF-α in the culture supernatants were measured by ELISA and the expression of mRNA for various molecules in monocytes was determined using RT-PCR. Flow cytometry analysis confirmed the bindings of anti-Sm mAb and anti-RNP mAb on viable human monocytes. Both anti-Sm mAb and anti-RNP mAb significantly increased the production of IL-6 and TNF-α of human monocytes in a dose-dependent manner, although the latter was more potent than the former. Of note, anti-Sm mAb synergistically enhanced the production and mRNA expression of IL-6 and TNF-α of human monocytes in the presence of anti-RNP mAb. Notably, anti-RNP mAb, but not anti-Sm mAb, significantly enhanced the mRNA expression of RelA in human monocytes. Finally, anti-Sm mAb still up-regulated the IL-6 production of monocytes in the presence of anti-RNP mAb under the influence of N-acetyl cysteine or pyrrolidine dithiocarbamate that totally abrogated the IL-6 production provoked by anti-Sm mAb alone in the absence of anti-RNP mAb. These results demonstrate that anti-Sm and anti-RNP antibodies synergistically up-regulate the expression of IL-6 and TNF-α in human monocytes. The data also suggest that the effect of anti-Sm in the synergy with anti-RNP might not involve NFkB activation.

## Introduction

Anti-RNP antibodies (anti-RNP) have been found to be expressed in systemic lupus erythematosus (SLE) as well as in mixed connective tissue disease (MCTD) which is frequently associated with pulmonary artery hypertension [1]. Previous studies demonstrated that anti-RNP bound human pulmonary artery endothelial cells (HPAECs) [2]. Accordingly, anti-RNP up-regulated the expression of adhesion molecules, including intercellular adhesion molecule-1 (ICAM-1), E-selectin and Class II molecules on HPAECs [3]. In addition, anti-RNP have been shown to enhance the production of proinflammatory cytokines, including IL-6 and TNF-α, by peripheral blood monocytes [4]. It is thus possible that anti-RNP might also bind human peripheral blood monocytes.

Anti-Sm antibodies (anti-Sm) are directed against proteins that constitute the common core of small nuclear ribonucleoprotein (snRNP) particles and are specifically expressed in patients with SLE [5]. Serum anti-Sm have been found to be associated with organic brain syndrome or acute confusional state (ACS) of diffuse neuropsychiatric SLE (NPSLE) [6,7]. Moreover, recent studies have disclosed that Q albumin, an indicator of blood-brain barrier (BBB) damages, was significantly correlated with serum anti-Sm in patients with NPSLE [8]. Of note, previous study showed that BBB damages were strongly linked to elevated levels of pro-inflammatory cytokines such as tumor necrosis factor-α (TNF-α) and interleukin-6 (IL-6) [9–11]. Thus, human monocytes may contribute to BBB damages through the production of pro-inflammatory cytokines [11]. It is therefore possible that serum anti-Sm might have such proinflammatory effects that result in endothelial dysfunction, leading to BBB dysfunction.

Notably, the expression of anti-Sm is always associated with anti-RNP in patients with SLE, although its mechanism remains uncertain [12]. Since anti-RNP bind HPAECs as well as human peripheral blood monocytes [2–4], it is also possible that anti-Sm might also bind these cells and influence the production of proinflammatory cytokines thereof. The present study was therefore designed in order to explore the effects of anti-Sm on the production of proinflammatory cytokines by human peripheral blood monocytes. Special attention was directed to the interactions of anti-Sm and anti-RNP in the effects on monocytes.

## Materials and methods

### Informed consents of the participants

Written informed consents were obtained from the participants of the study. This study was approved by the institutional ethical committee of Kitasato University School of Medicine (Ref. No. B09-55).

### Cell preparation

Human peripheral blood monocytes were purified by the same method as previously described [13]. Thus, peripheral blood mononuclear cells (PBMCs) were obtained from healthy adult volunteers who gave written informed consent by centrifugation of heparinized venous blood over sodium diatrizoate-Ficoll gradients. Monocytes were purified from PBMCs using Monocyte Isolation Kit II (Miltenyi Biotec, Tokyo, Japan). The monocytes contained >95% CD14+ monocytes, <0.1% CD3+ T cells, and <0.1% CD19+ B cells, as determined by analysis with flow cytometry [13].

### Antibodies

Monoclonal anti-Sm antibody (murine IgG3) (anti-Sm mAb), which recognizes BB´ and D proteins of Sm, was purchased from Thermo Scientific, Fremont, CA. Monoclonal anti-RNP antibody (murine IgG1) (anti-RNP mAb), which recognizes 68-kd protein of U1-RNP, was purchased from Synaptic systems, Gottingen, Germany. Murine control IgG1 and IgG3 were purchased from Cappel, West Chester, PA, and from Abcam, Cambridge, UK, respectively.

### Preparation of human IgG

Sera were obtained from 4 patients with SLE or MCTD or from 2 healthy individuals who gave written informed consent. IgG fractions were purified from the sera using a protein G–Sepharose 4FF column (Amersham Pharmacia Biotech, Uppsala, Sweden). The concentrations of anti-DNA antibodies (anti-DNA), anti-Sm and anti-RNP in the purified IgG were determined by ELISA using MESACUP (MBL, Nagoya, Japan). Since it has been shown that anti-ribosomal P antibodies enhance the IL-6 production of monocytes [13], the concentrations of anti-ribosomal P antibodies activity were determined by ELISA as previously described [14]. All the IgG samples had no detectable anti-ribosomal P antibodies. IgG fractions from patients or from healthy individuals were appropriately mixed to generate IgG preparations which contain varying concentrations of anti-Sm with the constant concentration of anti-RNP in the absence of anti-ribosomal P antibodies (Table 1).

**Table 1:** Preparation of mixtures of IgG containing varying concentrations of anti-Sm and anti-RNP.

	Contents of various IgG samples^a^							Concentrations		
No.	PtA (𝛍l)	PtB (𝛍l)	PtC (𝛍l)	PtD (𝛍l)	HE (𝛍l)	HF (𝛍l)	PBS (𝛍l)	Anti- RNP (U/ml)	Anti- Sm (U/ml)	IgG (mg/ml)
**1**	0	0	0	50	100	100	150	9	0	1.08
**2**	40	0	0	0	150	150	60	9	3	1.06
**3**	0	40	0	0	133	133	100	9	7	0.98
**4**	40	0	200	0	50	50	60	9	9	1.03
**5**	0	40	133	0	67	67	100	9	11	1.04
**6**	0	0	133	0	133	133	0	0	11	0.97
**7**	0	0	0	0	200	200	0	0	0	1.01

^a^IgG fractions from 4 patients (PtA, PtB, PtC, PtD) and from 2 healthy individuals (HE, HF) were mixed with or without phosphate buffered saline (PBS) as indicated. The concentrations of IgG, anti-Sm and anti-RNP in each IgG sample:

PtA (Patient A) (IgG; 2.69 mg/dl, Anti-Sm; 32 IU/ml, Anti-RNP; 96 IU/ml)

PtB (Patient B) (IgG; 3.58 mg/dl, Anti-Sm; 75 IU/ml, Anti-RNP; 96 IU/ml)

PtC (Patient C) (IgG; 1.18 mg/dl, Anti-Sm; 38 IU/ml, Anti-RNP; 0 IU/ml)

PtD (Patient D) (IgG; 4.28 mg/dl, Anti-Sm; 0 IU/ml, Anti-RNP; 77 IU/ml)

HE (Healthy E) (IgG; 1.10 mg/dl), HF (Healthy F) (IgG; 1.08 mg/dl).

### Reagents

Human IgG F(ab’)_2_ fragments (Gamma Venin P) were purchased from Sanofi, Paris, France. N-acetyl cysteine (NAC) (Sigma, St Louis, MO), pyrrolidine dithiocarbamate (PDTC) (Abcam), TPCA-1 (Abcam), methyl-β-cyclodextrin (Sigma) and cytochalasin D (Wako, Osaka, Japan) were also purchased. Human BD Fc Block was purchased from BD Biosciences, Franklin Lakes, NJ.

### Culture medium

RPMI 1640 medium (Nikken, Kyoto, Japan) supplemented with penicillin G (Life Technologies, Gaithersburg, MD) (100 U/ml), streptomycin (Life Technologies) (100 μg/ml), L-glutamine (Sigma) (0.3 mg/ml), and 10% fetal bovine serum (FBS) (Life Technologies) was used throughout the cultures.

### Flow cytometry

Fresh or cultured monocytes (5×10^5^/sample) were stained with anti-RNP mAb, anti-Sm mAb, IgG1 or IgG3 (5 μg/ml), followed by counterstaining with fluorescein isothiocyanate (FITC)-conjugated goat F(ab′)_2_ anti-mouse IgG (Cappel), as previously described [13]. The staining procedures were carried out at 4°C with staining buffer containing 2% normal human serum to block Fc gamma receptor [13]. After staining, the cells were treated in saline with 50 μg/ml propidium iodide (PI; Sigma) for more than 5 minutes at room temperature, followed by analysis using Cell Lab Quanta SC (Beckman Coulter, Miami, FL). The gating threshold for PI staining to identify viable cells was determined using live cells without PI staining. The density of staining was expressed as the change in mean fluorescence intensity (MFI) for staining of all the cells with anti-RNP or anti-Sm, which was calculated by subtracting the MFI of staining of all the cells with control IgG1 or IgG3, respectively. Also differences of MFI for staining with anti-RNP mAb from staining with control IgG1 and those of MFI for staining with anti-Sm mAb from staining with control IgG3 were statistically analyzed with Paired t test.

In some experiments, purified monocytes (1×10^6^/well) were cultured in 24-well microtiter plates (Nunc, Roskilde, Denmark) in culture medium at 37°C in 5% CO_2_ with the presence of anti-RNP mAb, anti-Sm mAb, IgG1 or IgG3 (5 μg/ml) for 24 hours, after which the cells were harvested and stained with FITC-conjugated goat F(ab′)_2_ anti-mouse IgG, followed by analysis on flowcytometry.

### Cell cultures

Purified monocytes (1×10^6^/well) were cultured in 24-well microtiter plates (Nunc) with anti-RNP mAb, anti-Sm mAb, control IgG1, or control IgG3 at various concentrations. In some experiments, purified monocytes were cultured in the presence of 100 μg/ml of human IgG preparations containing various concentrations of anti-Sm and anti-RNP (Table 1). After 48 or 96 hours of incubation, the supernatants were harvested and assayed for IL-6 and TNF-α using the Human IL-6 ELISA Kit (Affymetrix, San Diego, CA) and the TNF-α Human ELISA Kit High Sensitivity (Thermo Scientific), respectively.

### RNA isolation and real-time quantitative polymerase chain reaction (PCR)

After 4 hours of incubation, monocytes were harvested for RNA extraction. Total RNA was isolated from cultured cells using ISOGEN (Nippon Gene, Tokyo, Japan) according to the manufacturer's directions. cDNA was prepared from 1 μg of total RNA using PrimeScript II RTase (Takara Bio, Shiga, Japan) with Oligo dT primers (Takara Bio), and was subjected to analysis with real-time PCR using LightCycler 4.1 (Roche Diagnostics, Lewes, UK). Real-time PCR for IL-6, TNF-α and β-actin was performed using SYBR Premix Ex Taq II (Takara Bio) as previously described [13,15]. Amplification was performed according to the standard protocol recommended by the manufacturer. All results for the copy number of IL-6 mRNA or TNF-α mRNA were calibrated to the copy number of β-actin obtained from the same cDNA samples. Real-time PCR for NFkB1 (p50), NFkB2 (p52) and RelA (p65) in cultured monocytes was also performed as previously described [16]. The expression of mRNA for NFkB1 (p50), and RelA (p65) is shown as the ratio of the copy numbers to those of β-actin mRNA.

### Statistical analysis

Comparisons among 4 groups and between 2 groups were carried out by Repeated–Measures one-way ANOVA with multiple comparison and by Paired t test, respectively, using GraphPad Prism 7.03 (GraphPad Software, Inc., San Diego, CA, USA).

## Results

### Binding of anti-RNP mAb and anti-Sm mAb to human peripheral blood monocytes

**Fig 1:**
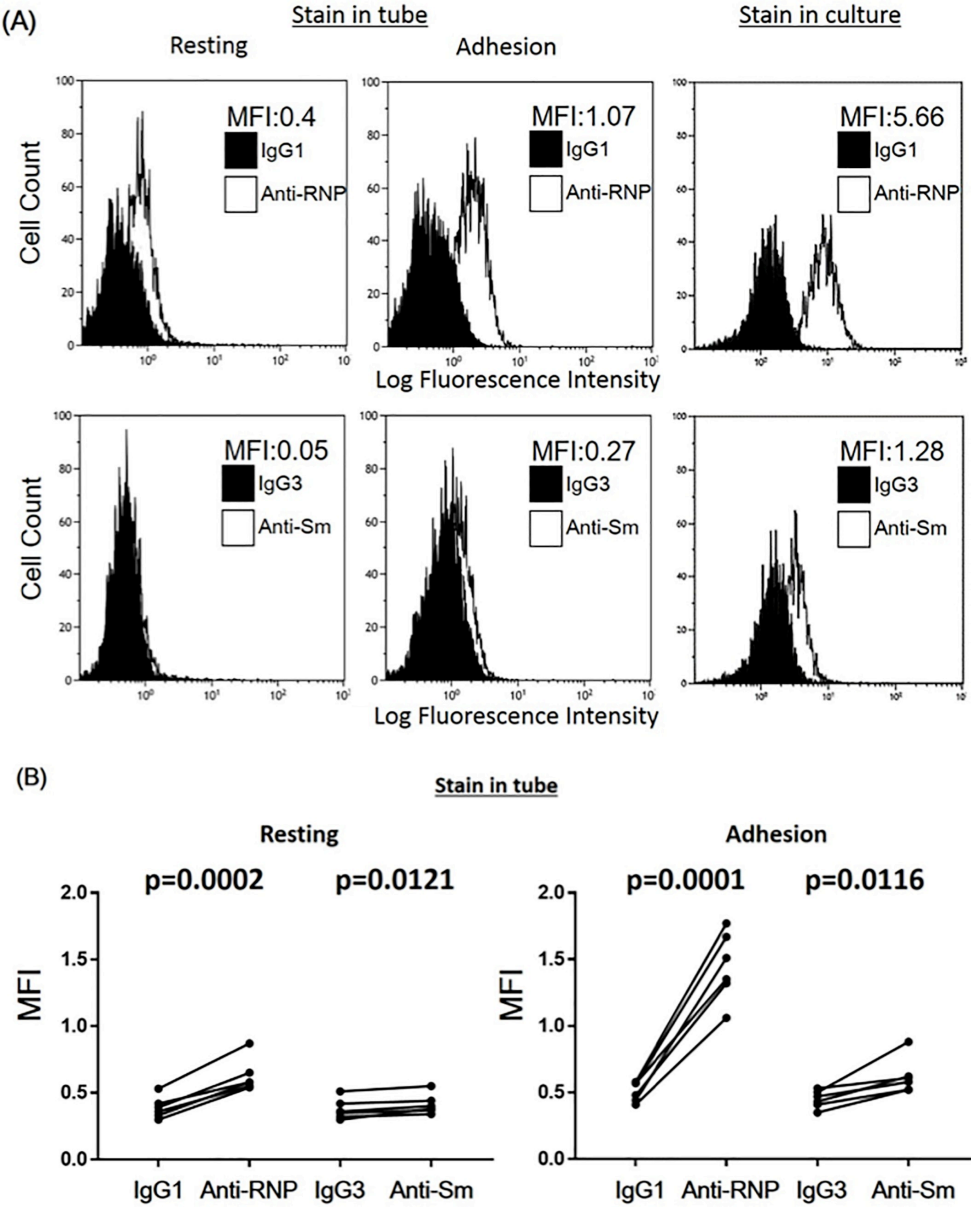
Flow cytometric analysis of binding of anti–Sm mAb and anti-RNP mAb to human peripheral blood monocytes. (**A)** Bindings of anti-Sm mAb and anti-RNP mAb on resting monocytes and monocytes activated by adhesion (stained in tubes), and binding of anti-Sm mAb and anti-RNP mAb during cultures. The mean fluorescence intensity (MFI) for specific anti-Sm mAb staining and anti-RNP mAb staining is indicated. Representative of 6 different experiments with similar results. (**B)** Differences of the mean fluorescence intensity (MFI) for control IgG1 vs anti-RNP mAb and control IgG3 vs anti-Sm mAb, in staining on resting monocytes or on monocytes activated by adhesion. Statistical significance was determined by paired t test.

Initial experiments were carried out to explore whether anti-RNP and anti-Sm might bind peripheral blood monocytes. As shown in Fig 1A, anti-RNP mAb bound to resting monocytes and more prominently to activated monocytes that had been cultured on flat-bottomed plastic plates for 24 hours [17]. Thus, the MFI for anti-RNP was significantly higher than that for IgG1(Fig 1B). Although the binding of anti-Sm mAb on the surface of resting monocytes were very modest compared with that of anti-RNP mAb, the MFI for anti-Sm was significantly higher than that for IgG3 (Fig 1B). However, after activation through adherence on flat-bottomed plastic plates for 24 hours, the binding of anti-Sm mAb on the surface of monocytes was significantly up-regulated. These results indicate that both anti-Sm and anti-RNP bind to the surface of viable human monocytes and that such binding is upregulated upon activation of human monocytes. Of note, further increased binding of anti-Sm and anti-RNP was found when monocytes were cultured with the presence of these antibodies (Fig 1A). Therefore, the data confirm that anti-Sm and anti-RNP bind monocytes to influence their functions.

### Enhancement of the production of IL-6 and TNF-αof human peripheral blood monocytes by anti-Sm mAb and anti-RNP mAb

**Fig 2:**
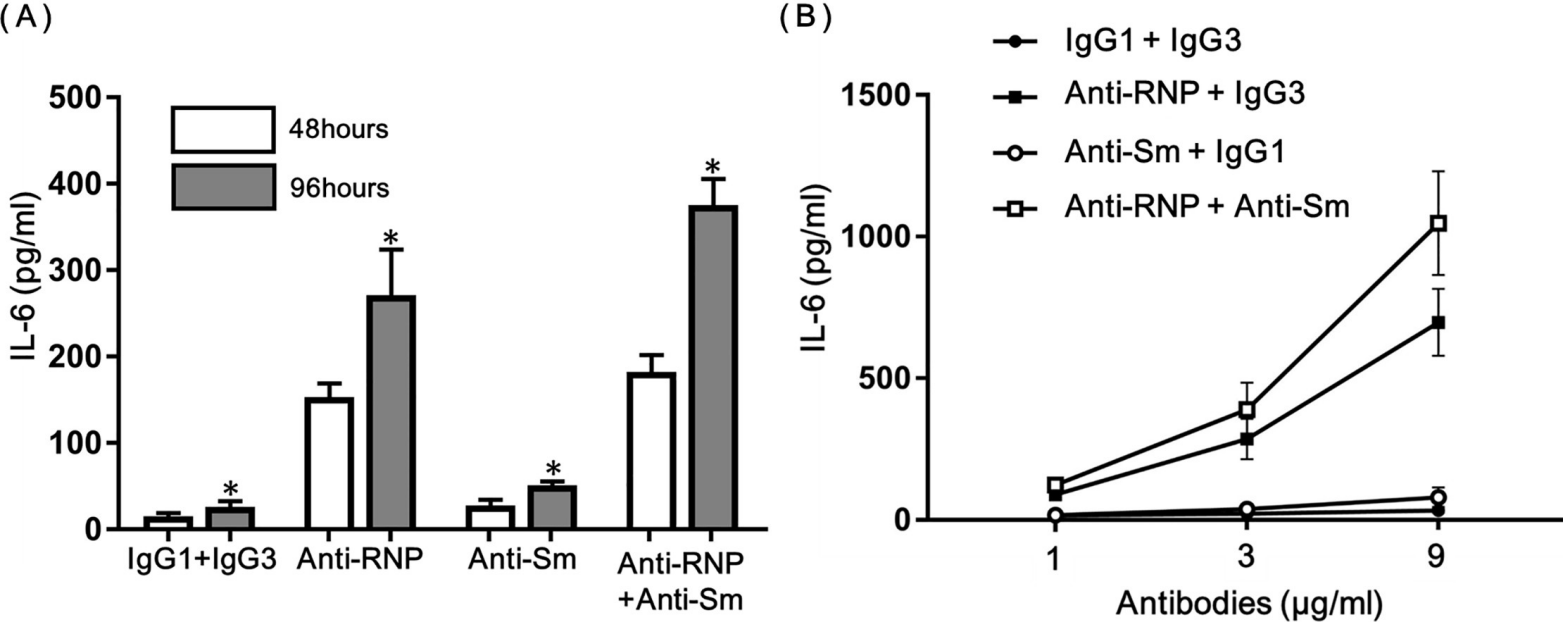
Effects of anti-RNP mAb and anti-Sm mAb on the production of IL-6 of peripheral blood monocytes. (**A)** Time kinetics of the effects of anti-RNP mAb and anti-Sm mAb. Highly purified monocytes were cultured in the presence of various antibodies (3 μg/ml). Mean values with standard deviation (Error bars) of 6 different experiments are shown. Statistical significance was examined by Paired t test. * Significant at p≤0.05 compared with 48 hours of culture. **(B)** Dose responses of anti-RNP mAb and anti-Sm mAb on the production of IL-6. Highly purified monocytes were cultured in the presence of various concentrations of anti-Sm mAb, anti-RNP mAb, control IgG1 or control IgG3. After 48 hours of incubation, the supernatants were assayed for IL-6. Mean values with standard deviation (Error bars) of 6 different experiments are shown.

Next experiments examined whether anti-Sm and anti-RNP might influence the function of human monocytes. Because the production of inflammatory cytokines is an important feature of monocytes, the effects of anti-Sm mAb and anti-RNP mAb on the production of IL-6 and TNF-αwere explored. As shown in Fig 2A, the production of IL-6 of human monocytes increased in a time-dependent manner from 48 hours to 96 hours. More importantly, anti-Sm mAb (3 μg/ml) as well as anti-RNP mAb (3 μg/ml) enhanced the production of IL-6 at each time point compared with that in control cultures with IgG1+IgG3, although the effect of the former was less marked than that of the latter. In addition, anti-Sm mAb further increased the production of IL-6 in the presence of anti-RNP mAb.

As shown in Fig 2B, anti-Sm mAb and anti-RNP mAb enhanced the production of IL-6 of monocytes in a dose-response manner. It appears that anti-Sm mAb further increased the production of IL-6 even in the presence of the higher concentration of anti-RNP mAb (9 μg/ml). These results suggest that the effects of anti-Sm might be synergistic with those of anti-RNP.

**Fig 3:**
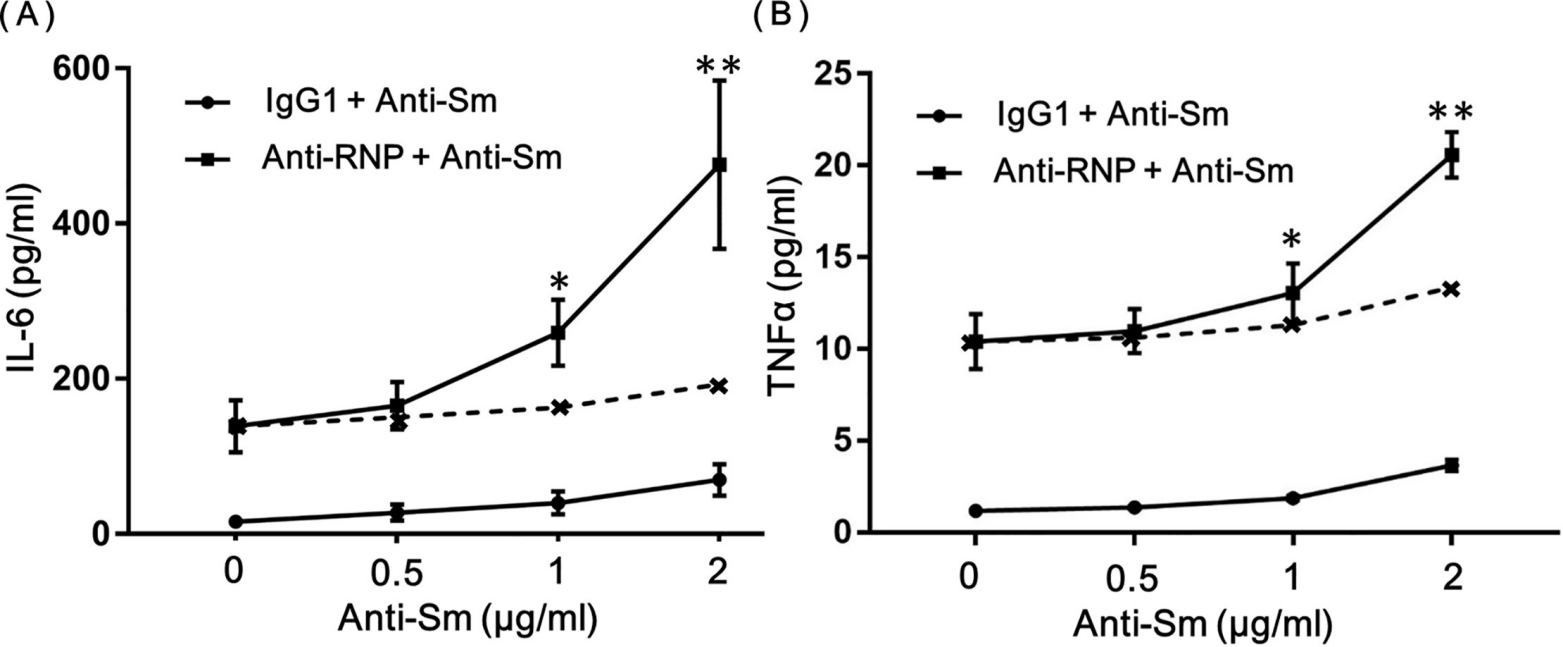
**Synergistic effects of anti-Sm mAb and anti-RNP mAb on the production of IL-6 (A) and TNF-α (B) of peripheral blood monocytes**. Highly purified monocytes were cultured with varying concentrations of anti-Sm mAb in the presence of anti-RNP mAb (1 μg/ml) or control IgG1 (1 μg/ml). After 48 hours of incubation, the supernatants were assayed for IL-6. Mean values with standard deviation (Error bars) of 5 different experiments are shown. The broken line indicate the sum of the IL-6 and TNF-α production in the presence of anti-RNP mAb alone (1 μg/ml) plus that in the presence of IgG1 (1 μg/ml) with various concentrations of anti-Sm mAb. Statistical significance was analyzed using Paired t test. * Significantly higher (p≤0.05) than the point of anti-Sm of 1 μg/ml in the broken line. * * Significantly higher (p≤0.005) than the point of anti-Sm of 2 μg/ml in the broken line.

To further explore the interactions between anti-Sm mAb and anti-RNP mAb in the regulation of the production of IL-6 and TNF-αof human monocytes, highly purified monocytes were cultured with various concentrations of anti-Sm (0–2 μg/ml) in the presence of the constant concentration of anti-RNP mAb or control IgG1 (1 μg/ml). As shown in Fig 3, anti-Sm mAb enhanced the production of IL-6 and TNF-αof human monocytes in a dose-dependent manner in the presence or absence of anti-RNP mAb. More importantly, it was evident that the effects of anti-Sm mAb were synergistic with those of anti-RNP mAb. Thus, the production of IL-6 and TNF-αin the presence of both anti-Sm mAb and anti-RNP mAb (solid line) apparently exceeded the sum of the production of IL-6 and TNF-αin the presence of anti-Sm mAb alone plus that in the presence anti-RNP mAb alone (broken line). The results confirm that anti-Sm mAb and anti-RNP mAb exert synergistic enhancing effects on the production of IL-6 and TNF-αof human monocytes.

### Enhancement of the production of IL-6 of human peripheral blood monocytes by human anti-Sm and anti-RNP

**Fig 4:**
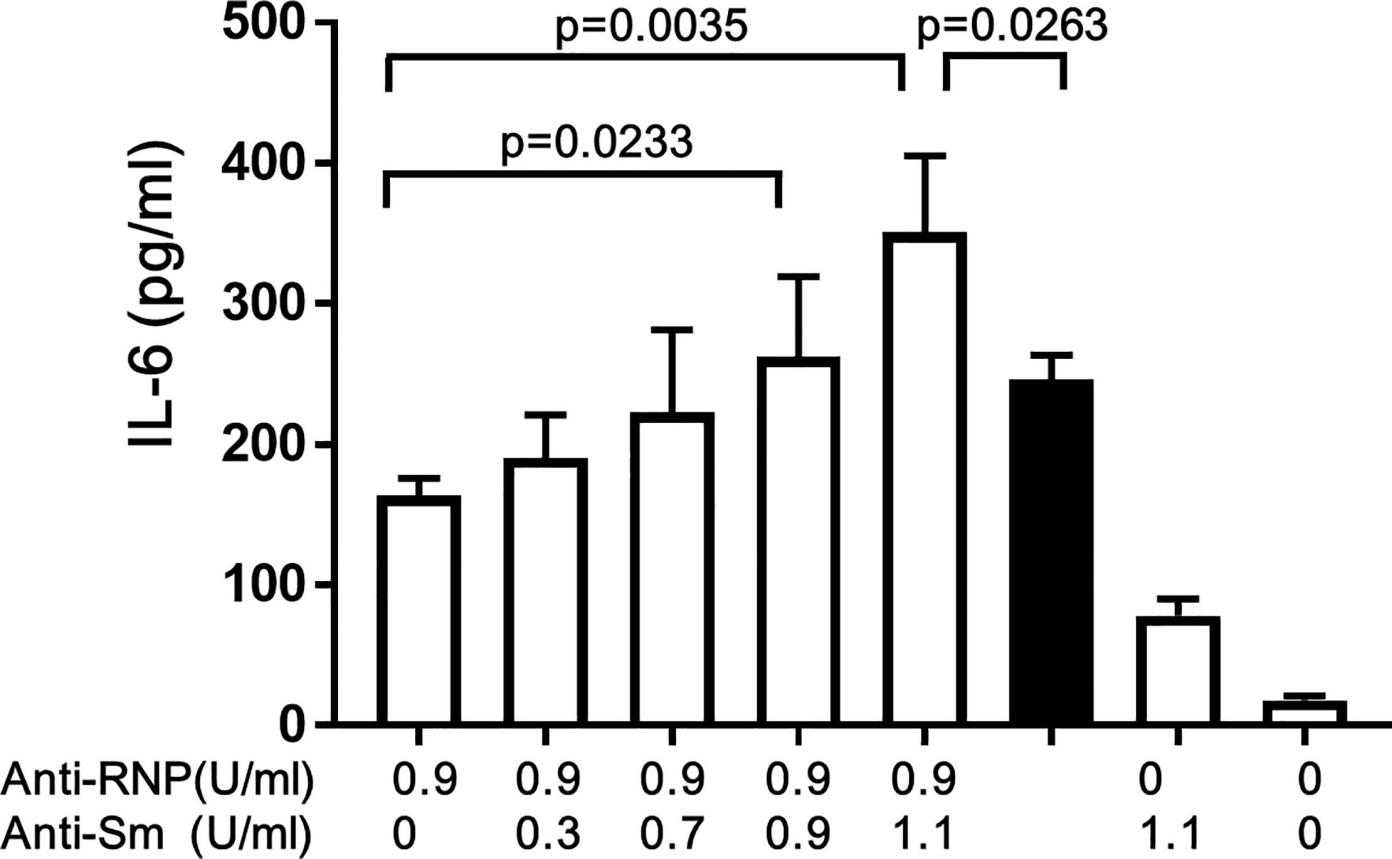
Effects of human anti-Sm and human anti-RNP on the production of IL-6 of peripheral blood monocytes. Highly purified monocytes (1×10^6^/ml) from 4 healthy individuals were cultured in the presence of various IgG fractions containing varying concentrations of human anti-Sm and human anti-RNP at a final concentration of 100 μg/ml IgG. Thus, each IgG preparation shown in Table 1 was added at 1:10 [volume/volume]. After 48 hours of incubation, the supernatants were assayed for IL-6. Mean values with standard deviation (Error bars) of 4 different experiments are shown. The solid bar indicate the sum of IL-6 production in the presence of anti-RNP (0.9 U/ml) alone plus that in the presence of anti-Sm (1.1 U/ml) alone. Statistical significance was analyzed using Paired t test.

Next experiments were performed to confirm that human anti-Sm and human anti-RNP exert comparable effects on the production of IL-6 to those by murine anti-Sm mAb and murine anti-RNP mAb. As shown in Fig 4, the production of IL-6 was increased along with the increase in concentrations of anti-Sm in the presence of the constant concentration of anti-RNP. Again, the IL-6 production in the presence of both human anti-Sm and human anti-RNP was significantly higher than the sum of the IL-6 production in the presence of anti-RNP (0.9 U/ml) alone plus that in the presence of anti-Sm (1.1 U/ml) alone (solid bar in Fig 4). The results confirm that human anti-Sm and human anti-RNP synergistically enhance the production of IL-6 of human peripheral blood monocytes.

### Effects of Fc receptor blocking on the synergistic enhancement of IL-6 production of peripheral blood monocytes by anti-Sm mAb and anti-RNP mAb

**Fig 5:**
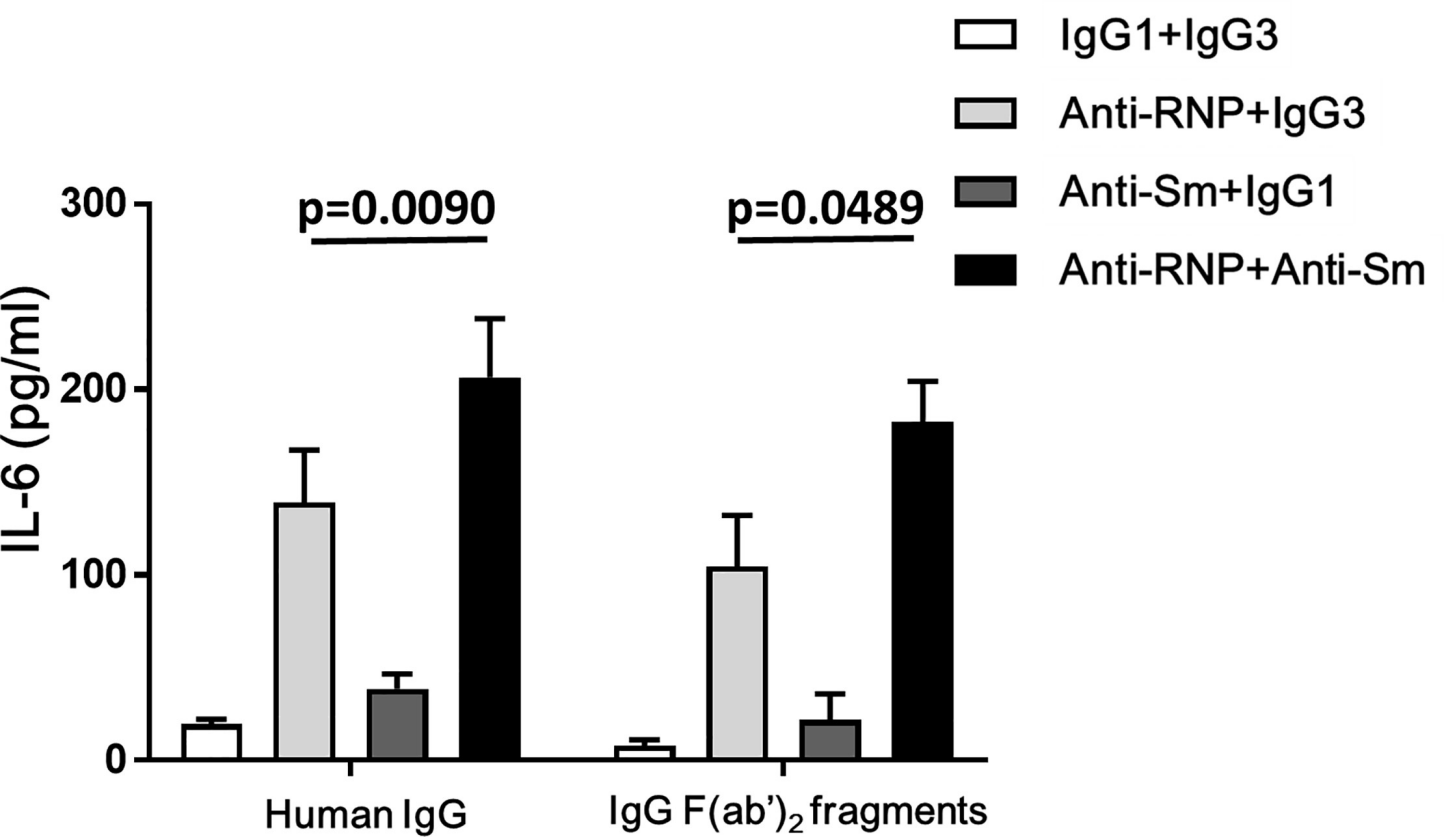
Effects of human IgG or human IgG F(ab’)_2_ fragments on the synergistic enhancement of the production of IL-6 of peripheral blood monocytes by anti-Sm mAb and anti-RNP mAb. Highly purified monocytes were cultured in the presence of whole molecule human IgG (100 μg/ml) or human IgG F(ab')_2_ fragments (100 μg/ml) with various combination of anti-Sm mAb, anti-RNP mAb, control IgG1 or IgG3 (3 μg/ml). After 48 hours of incubation, the supernatants were assayed for IL-6. Mean values with standard deviation (error bars) of 3 different experiments are shown. Statistical significance was evaluated by paired t test.

In order to examine whether Fc portions are required for the effects of anti-Sm mAb and anti-RNP mAb, experiments were carried out in which the effects of addition of whole molecule human IgG and human IgG F(ab')_2_ fragments were explored. As shown in Fig 5, the synergistic effects of anti-Sm mAb and anti-RNP mAb on the IL-6 production of monocytes in cultures with whole molecule human IgG (100 μg/ml) were comparable to those in cultures with human IgG F(ab')_2_ fragments (100 μg/ml). The results therefore suggest that Fc portions might not be required for the synergistic effects of anti-Sm mAb and anti-RNP mAb.

**Fig 6:**
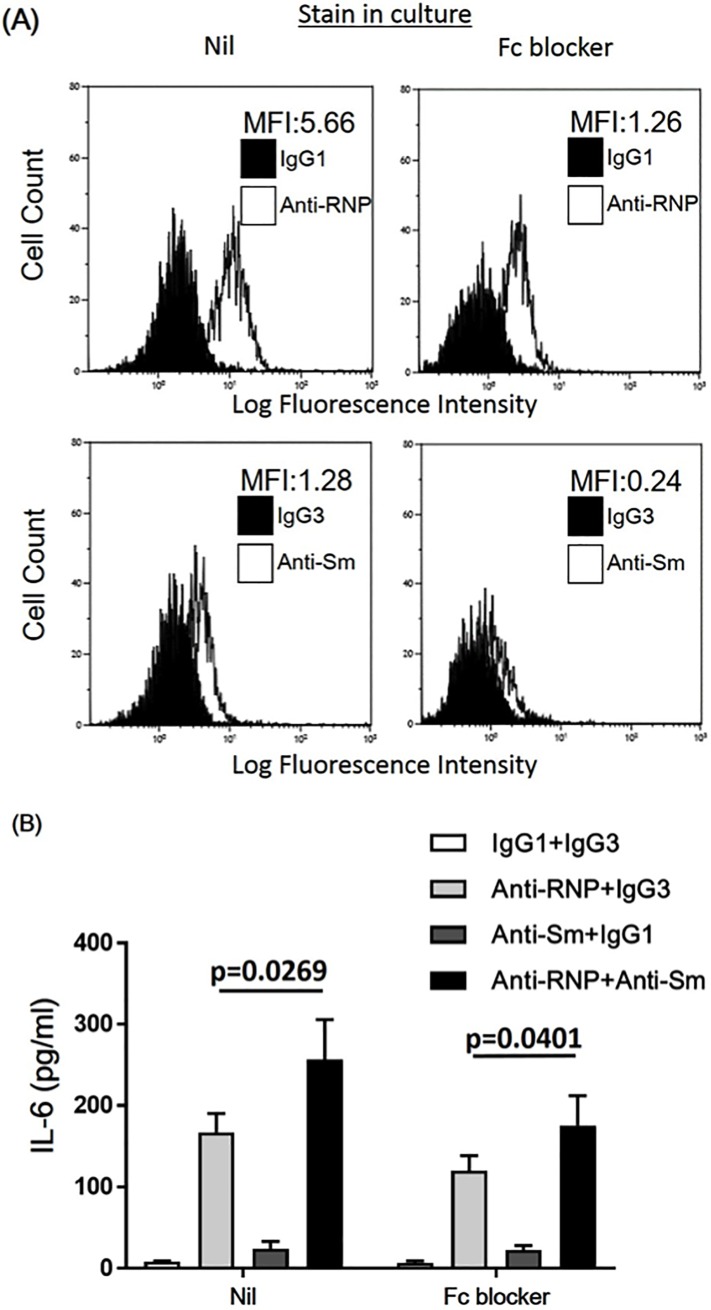
Effects of human recombinant Fc protein on the synergistic enhancement of the production of IL-6 of peripheral blood monocytes by anti-Sm mAb and anti-RNP mAb. Highly purified monocytes were cultured with various combination of anti-Sm mAb, anti-RNP mAb, control IgG1 or IgG3 (3 μg/ml) in the presence or absence of the human Fc blocker (Human BD Fc Block) (50 μg/ml). (A) After 24 hours the cells were harvested and stained with FITC-conjugated goat F(ab′)_2_ anti-mouse IgG, followed by analysis on flowcytometry. The mean fluorescence intensity (MFI) for specific anti-Sm mAb staining and anti-RNP mAb staining is indicated. Representative of 3 different experiments with similar results. (B) After 48 hours of incubation, the supernatants were assayed for IL-6. Mean values with standard deviation (error bars) of 3 different experiments are shown. Statistical significance was evaluated by paired t test.

Further experiments were carried out in which the effects of a purified recombinant Fc protein (Human BD Fc Block), which minimizes non-specific binding of immunoglobulins to Fc receptors, was examined. As shown in Fig 6A, addition of the human Fc blocker markedly decreased the binding of ant-Sm mAb and anti-RNP mAb on human monocytes. However, the synergistic effects of anti-Sm mAb and anti-RNP mAb on the IL-6 production of human monocytes were preserved in the presence of the human Fc blocker (Fig 6B). The results thus confirm that Fc portions are not required for the synergistic effects of anti-Sm mAb and anti-RNP mAb on the IL-6 production of human monocytes.

### Effects of anti-Sm mAb and anti-RNP mAb on the expression of mRNA for IL-6 and TNF-α in human peripheral blood monocytes

**Fig 7:**
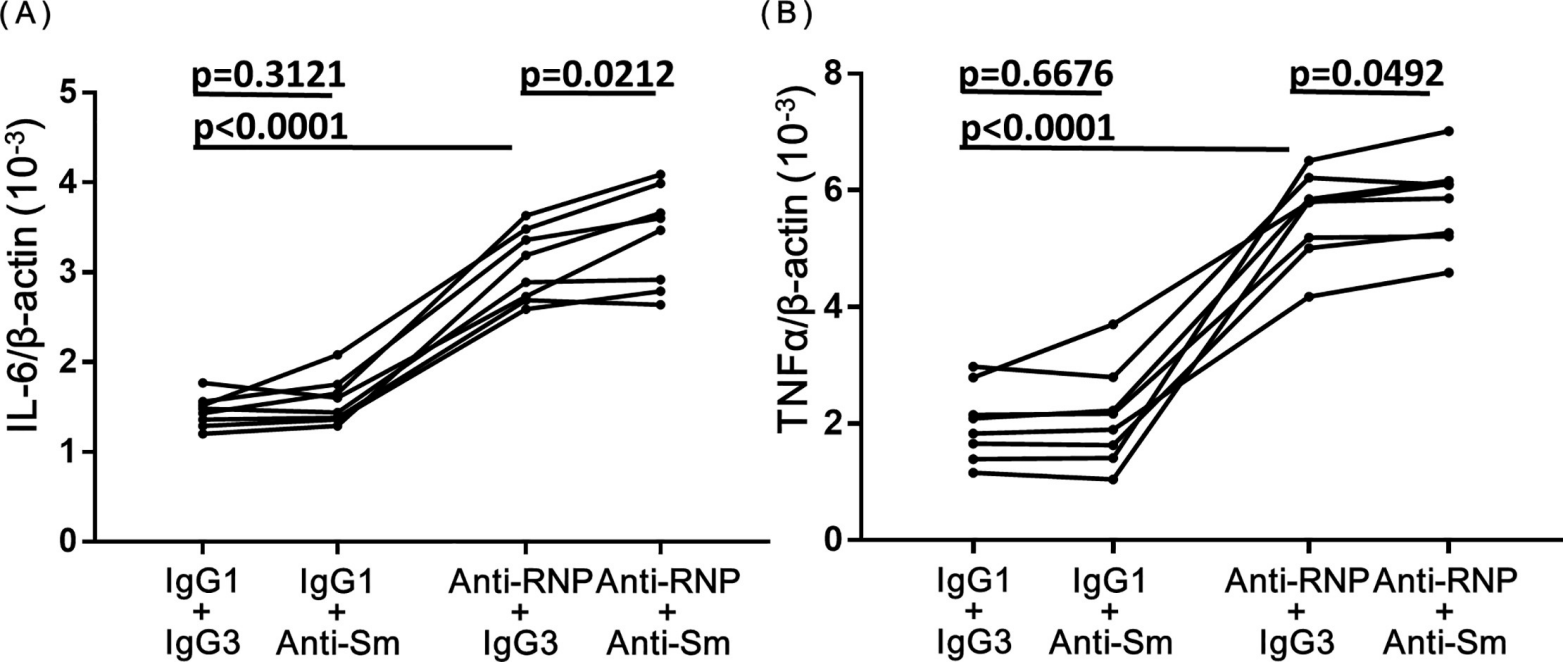
**Effects of anti-Sm mAb and anti-RNP mAb on the expression of mRNA for IL-6 (A) and TNF-α (B) in peripheral blood monocytes.** Highly purified monocytes from 8 healthy individuals were cultured with various combination of anti-Sm mAb, anti-RNP mAb, control IgG1 or IgG3 (3 μg/ml). After 4 hours of incubation, total RNA was isolated, and real-time quantitative polymerase chain reaction was performed as described in Materials and Methods. All results for IL-6 and TNF-α mRNA copy numbers were calibrated to the copy numbers of β-actin from the same cDNA sample. Statistical significance was evaluated by Repeated-Measures one-way ANOVA with multiple comparison.

Next experiments were carried out to explore whether the synergistic enhancing effects of anti-Sm mAb and anti-RNP mAb might be observed at mRNA levels. As shown in Fig 7, anti-Sm mAb (3 μg/ml) had no significant effect on the expression of mRNA for IL-6 and TNF-αin monocytes in the absence of anti-RNP mAb. However, anti-Sm mAb (3μg/ml) significantly increased the expression of mRNA for IL-6 and TNF-αin monocytes in the presence of anti-RNP mAb (3 μg/ml). The results indicate that the synergistic enhancing effects on the production of IL-6 and TNF-αof human monocytes by anti-Sm mAb and anti-RNP mAb are accounted for by their effects on the expression of mRNA for IL-6 and TNF-α.

### Effect of anti-Sm mAb and anti-RNP mAb on the expression of mRNAs for various components of NFkB in human peripheral blood monocytes

**Fig 8:**
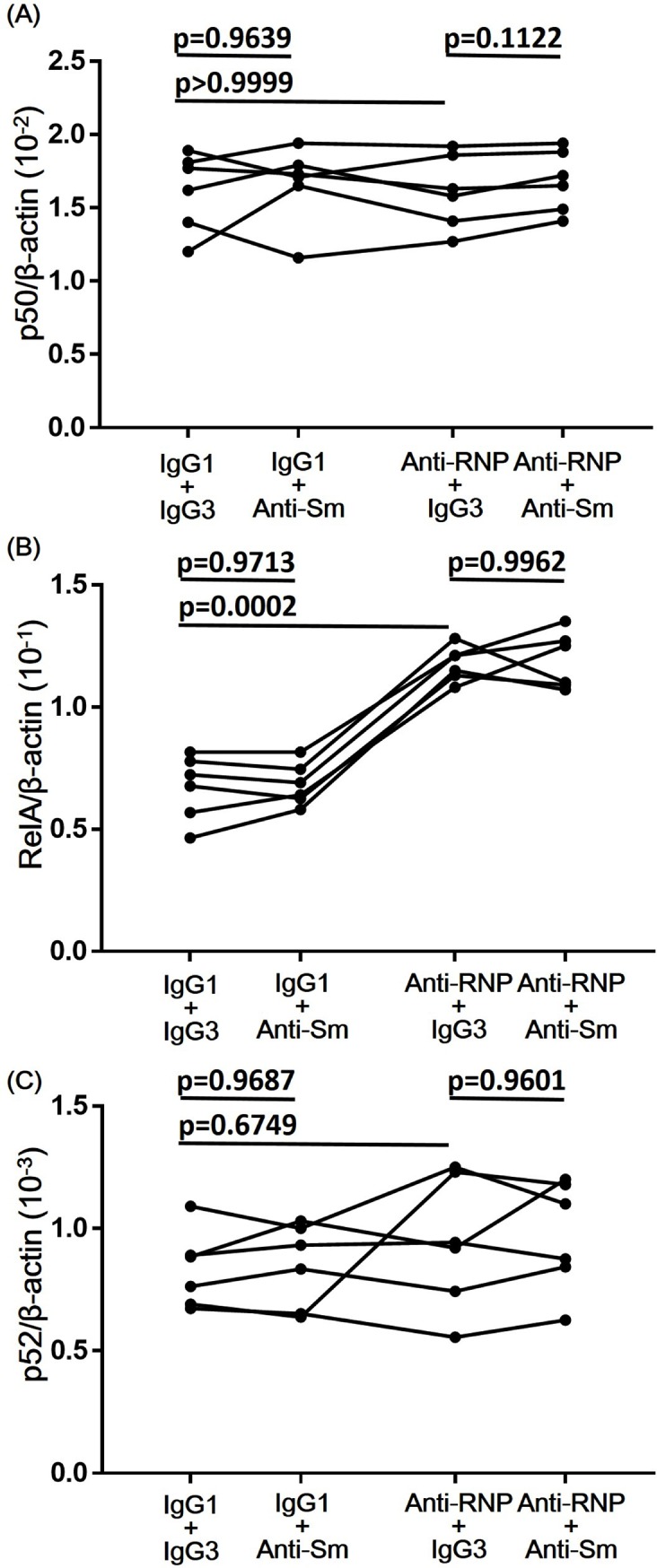
Effects of anti-Sm mAb and anti-RNP mAb on the expression of mRNAs for various components of NFkB. Highly purified monocytes from 6 healthy individuals were cultured with various combination of anti-Sm mAb, anti-RNP mAb, control IgG1 or IgG3 (3 μg/ml). After 4 hours of incubation, total RNA was isolated, and real-time quantitative polymerase chain reaction was performed as described in Materials and Methods. All results for NFkB1 (p50), RelA (p65) and NFkB2 (p52) mRNA copy numbers were calibrated to the copy numbers of β-actin from the same cDNA sample. Statistical significance was evaluated by Repeated-Measures one-way ANOVA with multiple comparison.

Previous studies have demonstrated that the enhancement of the expression of mRNAs for various components of NFkB results in the up-regulation of activity of NFkB [18,19]. Next experiments were carried out to explore the effects of anti-Sm mAb and anti-RNP mAb on the expression of mRNAs for various components of NFkB. Highly purified peripheral blood from healthy individuals were cultured in the presence of anti-Sm mAb and anti-RNP mAb for 4 hours, after which the expression of mRNA for various components of NFkB was examined. As shown in Fig 8, anti-RNP mAb, but not anti-Sm mAb, significantly enhanced the expression of RelA (p65) mRNA, whereas neither mAb significantly influenced the expression of NFkB1 (p50) or NFkB2 (p52). The results indicate that anti-RNP mAb, but not anti-Sm mAb, enhances the expression of RelA (p65) mRNA. In addition, it is suggested that the action of ant-Sm mAb in the synergism with anti-RNP mAb might involve such mechanisms other than p65 mRNA up-regulation.

**Fig 9:**
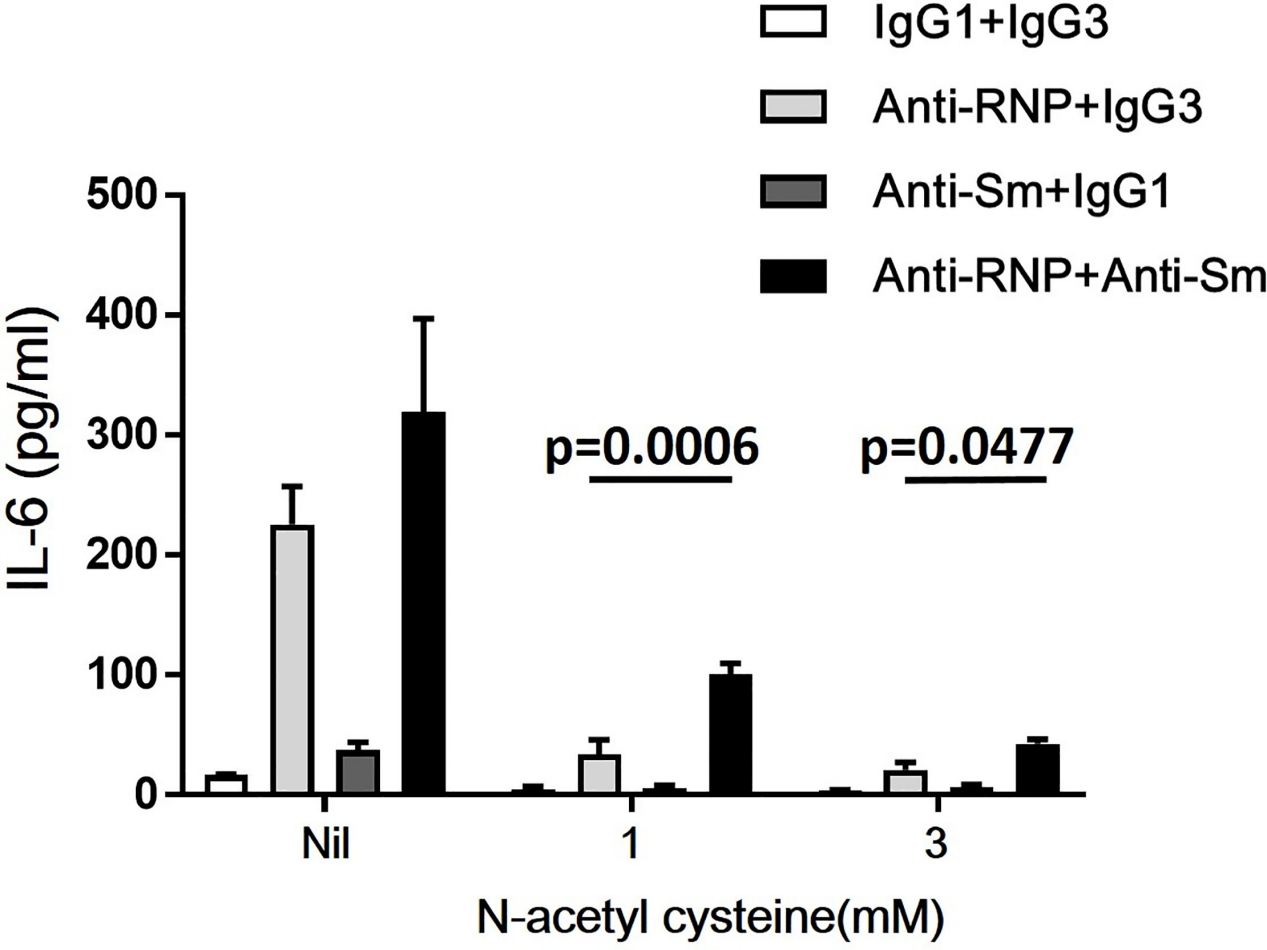
Effects of N-acetyl cysteine (NAC) on the synergistic enhancement of the production of IL-6 of peripheral blood monocytes by anti-Sm mAb and anti-RNP mAb. Highly purified monocytes were cultured in the presence or absence of NAC (1 mM or 3 mM) with various combination of anti-Sm mAb, anti-RNP mAb, control IgG1 or IgG3 (3 μg/ml). After 48 hours of incubation, the supernatants were assayed for IL-6. Mean values with standard deviation (error bars) of 3 different experiments are shown. Statistical significance was evaluated by paired t test.

**Fig 10:**
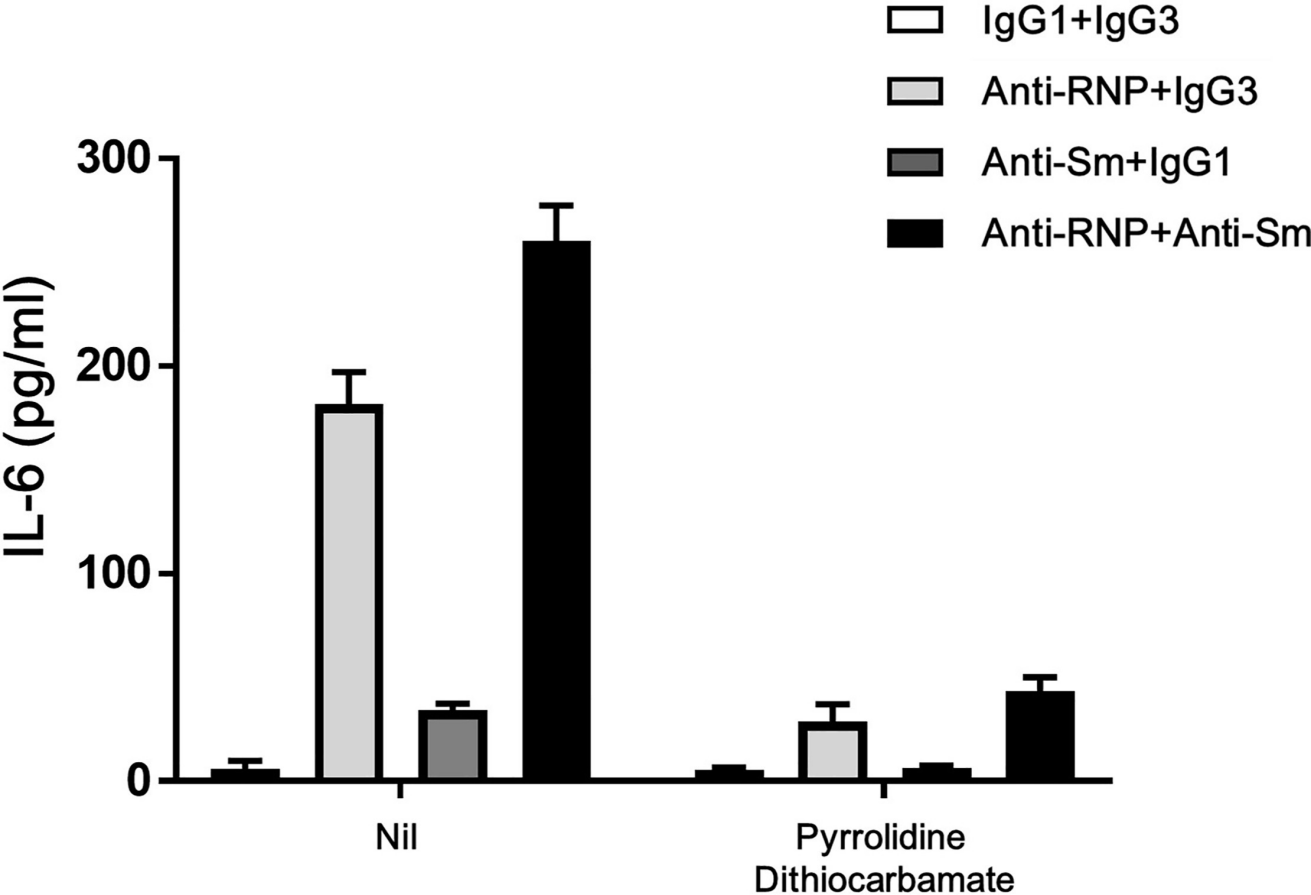
Effects of pyrrolidine dithiocarbamate (PDTC) on the synergistic enhancement of the production of IL-6 of peripheral blood monocytes by anti-Sm mAb and anti-RNP mAb. Highly purified monocytes were cultured in the presence or absence of PDTC (50 **𝛍**M) with various combination of anti-Sm mAb, anti-RNP mAb, control IgG1 or IgG3 (3 μg/ml). After 48 hours of incubation, the supernatants were assayed for IL-6. Mean values with standard deviation (error bars) of 2 different experiments with reproducible results are shown.

Activation of NFkB transcription factor is critical for the expression of proinflammatory cytokines in human monocytes [20,21]. To investigate the mechanism of synergistic enhancement of expression of IL-6 by anti-Sm mAb and anti-RNP mAb, next experiments examined the effects of NAC, an inhibitor of NFkB [22]. As shown in Fig 9, the enhancement of production of IL-6 of monocytes by anti-Sm mAb and anti-RNP mAb was markedly decreased by addition of NAC (1–3 mM). Thus, NAC markedly suppressed the baseline IL-6 production and almost completely abrogated the enhancing effect of anti-Sm mAb alone on IL-6 production in the absence of anti-RNP mAb. However, anti-Sm mAb still markedly up-regulated the IL-6 production in the presence of anti-RNP under the influences of NAC. It has been shown that the antioxidant PDTC specifically inhibits the transcription of IL-6, IL-8, and GM-CSF genes through the inhibition of the NFkB activation, while increasing the expression of AP-1 [23]. In the presence of PDTC (50 **𝛍**M), which suppressed the baseline IL-6 production, anti-Sm mAb still enhanced the IL-6 production in the presence of anti-RNP mAb (Fig 10). These results strongly suggest that the synergistic effects of anti-Sm mAb on the IL-6 production of monocytes in the presence of anti-RNP mAb might not involve the activation of NFkB.

### Effects of methyl-β-cyclodextrin or cytochalasin D on the synergistic enhancement of IL-6 production of peripheral blood monocytes by anti-Sm mAb and anti-RNP mAb

**Fig 11:**
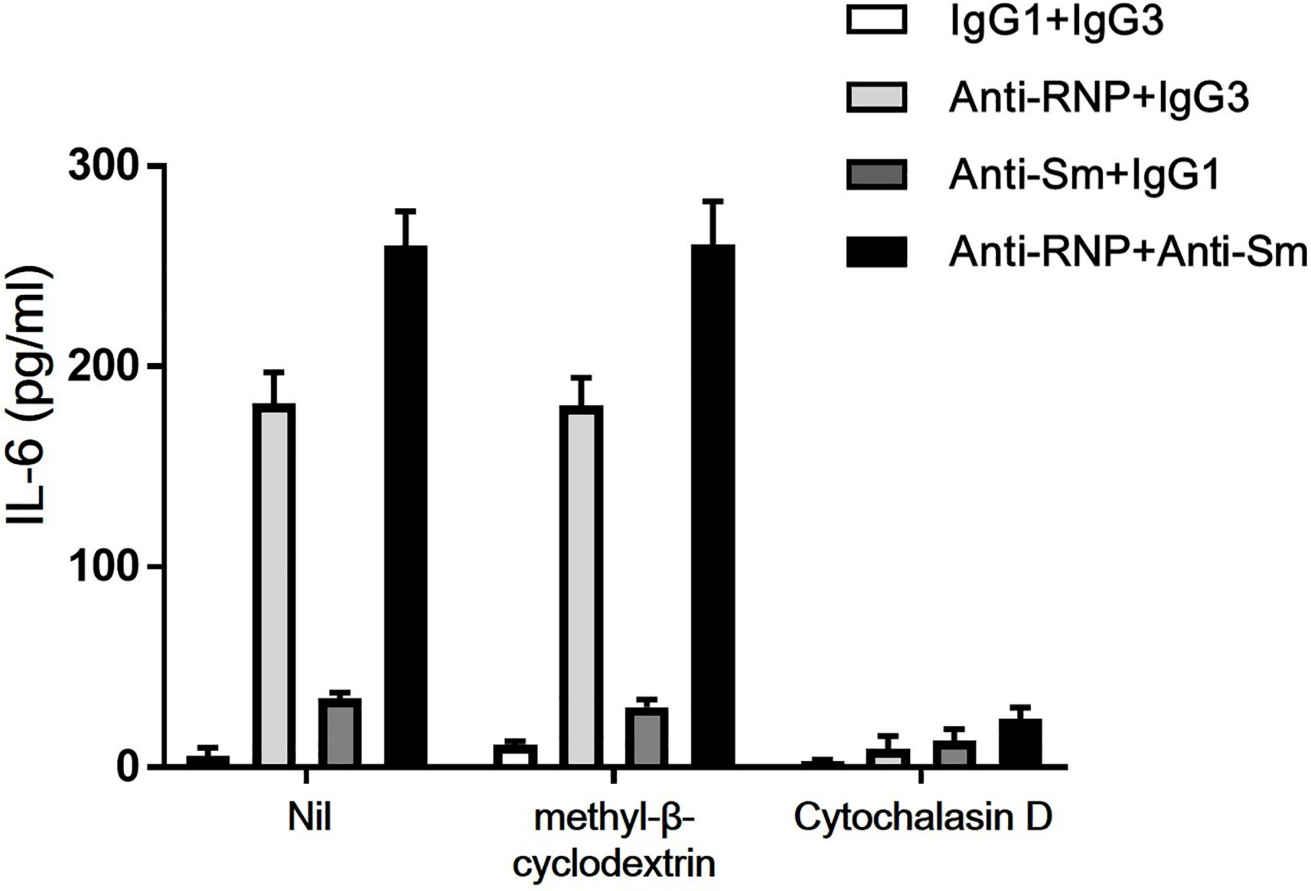
Effects of methyl-β-cyclodextrin and cytochalasin D on the synergistic enhancement of the production of IL-6 of peripheral blood monocytes by anti-Sm mAb and anti-RNP mAb. Highly purified monocytes were cultured in the presence or absence of methyl-β-cyclodextrin (5mM) and cytochalasin D (10 μM) with various combination of anti-Sm mAb, anti-RNP mAb, control IgG1 or IgG3 (3 μg/ml). After 48 hours of incubation, the supernatants were assayed for IL-6. Mean values with standard deviation (error bars) of 2 different experiments with reproducible results are shown.

In order to further explore the mechanisms of synergistic enhancement of IL-6 production of monocytes by anti-Sm and anti-RNP, the influences of various compounds were examined, including methyl-β-cyclodextrin (5 mM) and cytochalasin D (10 μM) affecting cell membrane lipid accumulation [24] and actin polymerization [25], respectively. As shown in Fig 11, methyl-β-cyclodextrin did not affect the effects of anti-Sm mAb and anti-RNP mAb on the IL-6 production of monocytes, whereas cytochalasin D almost completely abrogated it. The results indicate that the synergistic enhancement of the IL-6 production of monocytes by anti-Sm mAb and anti-RNP mAb does not involve membrane lipid accumulation, but requires actin polymerization.

## Discussion

Although anti-Sm and anti-RNP recognize nuclear proteins, several studies have shown that they bound the surface of certain types of cells. Thus, it has been shown that the epitopes recognized by anti-Sm existed on the surface of neuronal cells [7], while anti-RNP were found to bind endothelial cells [2]. The results in the current studies confirm that the epitopes recognized by anti-Sm and anti-RNP exist on the surface of human peripheral blood monocytes. Of note, the results also demonstrate that the expression of epitopes for anti-Sm and anti-RNP on the surface of human monocytes are upregulated upon activation. In this regard, the regulation of the expression of the epitopes for anti-Sm and anti-RNP was comparable to that of the expression of the epitopes for anti-ribosomal P antibodies in human monocytes [13].

The data in the present study have demonstrated that anti-Sm and anti-RNP enhanced the production of IL-6 and TNF-α of human monocytes at protein and mRNA levels, as is also the case in anti-ribosomal P antibodies [13]. The enhancing effect of anti-Sm alone was modest and much smaller than that of anti-RNP alone. However, anti-Sm enhanced the production of IL-6 and TNF-α of human monocytes much more markedly in the presence of anti-RNP. Apparently, the effects of anti-Sm and anti-RNP on the production of IL-6 and TNF-α were synergistic rather than additive.

The synergistic effects suggest that the signal delivered by anti-Sm might be different from that delivered by anti-RNP. In this regard, the data in the present study revealed that anti-RNP, but not anti-Sm, up-regulated the expression of RelA (p65) mRNA. Notably, the enhanced expression of RelA (p65) has been found to result in the activation of NFkB signaling [18,19]. Therefore, it is also possible that anti-RNP might also promote the activation of NFkB. Accordingly, NAC and PDTC markedly decreased the production of IL-6 in the presence of anti-RNP alone. However, anti-Sm still enhanced the IL-6 production in the presence of anti-RNP under the influences of NAC or PDTC which almost completely abrogated the IL-6 production by anti-Sm alone in the absence of anti-RNP. Moreover, TPCA-1, an IKK inhibitor [24], did not affect the synergy between anti-Sm and anti-RNP, either (S1 Fig). The data therefore indicate that the signals delivered by anti-Sm in the synergism with anti-RNP do not involve NFkB. Further studies are required to identify the nature of signals delivered by anti-Sm.

The results in the present study revealed that methyl-β-cyclodextrin did not affect the effects of anti-Sm and anti-RNP on the IL-6 production of monocytes, whereas cytochalasin D almost completely abrogated it. The results indicate that the synergistic enhancement of the IL-6 production of monocytes by anti-Sm and anti-RNP does not involve membrane lipid accumulation [25]. However, we showed that the synergistic enhancement of the IL-6 production by human monocytes requires intact actin filaments since this process was almost completely blocked by cytochalasin D [26]. Although the current studies partially characterized the mechanism of the synergistic enhancement of anti-Sm and anti-RNP of the activation of human monocytes, further studies are necessary to delineate the whole sequalae of the activation of monocytes by these autoantibodies.

Anti-Sm are directed against proteins that constitute the common core of small nuclear ribonucleoprotein (snRNP) particles [5]. Of note, all patients with positive serum anti-Sm express also serum anti-RNP in clinical practice [5]. Therefore, the synergistic effects of anti-Sm and anti-RNP demonstrated in the present study are considered to take place always in vivo and play a role in the development of inflammatory reactions in SLE. Anti-RNP react with proteins that are associated with U1-RNA and form U1snRNP, including A protein, C protein and 68-kd protein [5]. Anti-RNP mAb used in the present studies reacts only with 68-kd protein. It should be noted, however, that all human sera containing anti-U1-RNP antibodies reacted the cloned 68-kd RNP protein [12]. In fact, IgG fractions containing anti-RNP purified from the patients’ sera enhanced the production of IL-6 of monocytes in the present study. However, it remains to be elucidated whether antibodies to A protein or to C protein might have similar effects.

Previous studies demonstrated that serum anti-Sm were significantly higher in acute confusional state (ACS) than that in non-ACS diffuse neuropsychiatric SLE (NPSLE) or focal NPSLE [6,7]. Of note, serum IL-6 was also significantly higher in ACS than that in the other 2 groups of NPSLE [27]. Moreover, it has been found that serum anti-Sm as well as serum IL-6 levels were significantly correlated with Q albumin in patients with diffuse NPSLE, including ACS and non-ACS [8,27]. Notably, it was disclosed that the increased production of IL-6 in the CNS could influence the function of BBB [28]. On the other hand, it should be emphasized that TNF-α has been found to result in the damages of BBB [10,11]. Therefore, the results in the current studies support the hypothesis that anti-Sm might cause BBB damages through upregulation of the production of IL-6 and TNF-α.

## Conclusions

The present study has confirmed that anti-Sm as well as anti-RNP bind on the surface of human monocytes. More importantly, it has been demonstrated that anti-Sm and anti-RNP synergistically enhance the production of IL-6 by human monocytes. Although the present study partially disclosed the mechanism of synergistic effects by anti-Sm and anti-RNP, further studies would be important for a complete understanding of the roles of these antibodies in the pathogenesis of SLE.

## Supporting information

S1 Fig Effect of an IKK inhibitor TPCA-1 on the synergistic enhancement of the production of IL-6 of peripheral blood monocytes by anti-Sm mAb and anti-RNP mAb. Highly purified monocytes were cultured in the presence or absence of TPCA-1 (20, 40, 5000 nM) with various combination of anti-Sm mAb, anti-RNP mAb, control IgG1 or IgG3 (3 μg/ml). After 48 hours of incubation, the supernatants were assayed for IL-6. Mean values with standard deviation (error bars) of 2 different experiments with reproducible results are shown.
(DOCX)
