# Supplementary figures and images for: Synergistic enhancement of production of proinflammatory cytokines of human peripheral blood monocytes by anti-Sm and anti-RNP antibodies
Source: PLoS One. 2018 Dec 20;13(12):e0209282. doi: 10.1371/journal.pone.0209282 (PMC6301657; doi:10.1371/journal.pone.0209282)

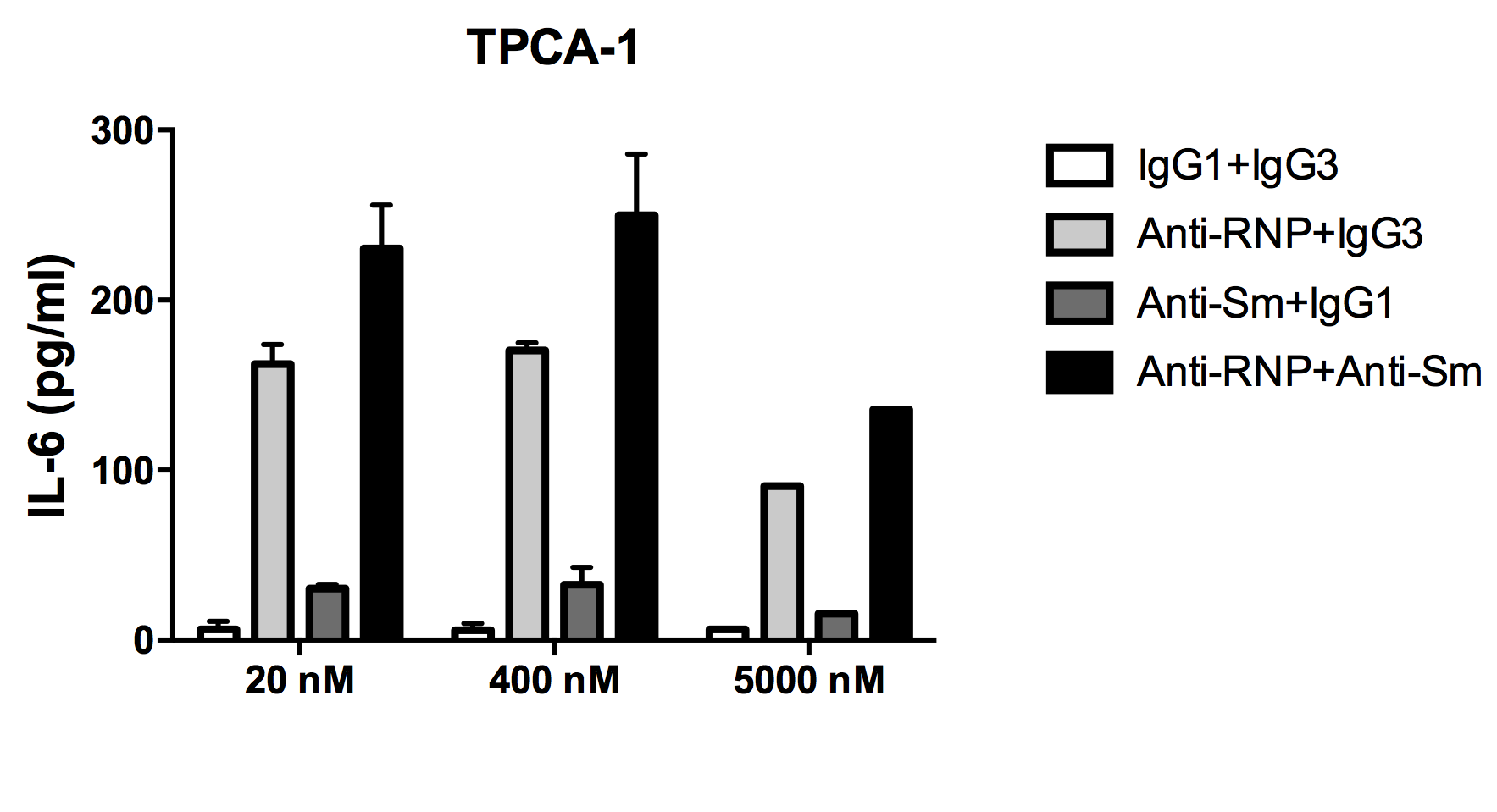

Supplement: S1 Fig — Highly purified monocytes were cultured in the presence or absence of TPCA-1 (20, 40, 5000 nM) with various combination of anti-Sm mAb, anti-RNP mAb, control IgG1 or IgG3 (3 μg/ml). After 48 hours of incubation, the supernatants were assayed for IL-6. Mean values with standard deviation (error bars) of 2 different experiments with reproducible results are shown. (DOCX) [file pone.0209282.s001.docx]
